# Materia Medica for Students—Biddle

**Published:** 1874-08

**Authors:** 


MATERIA MEDICA, for the use of Students. By John B. Biddle, M.D., Prof. of Materia Medica and General Therapeutics in Jefferson Medical College, etc., etc. Sixth edition, revised and enlarged, with illustrations. Philadelphia: Lindsay & Blakiston. 1874. Octavo. Cloth. Pp. 435. Price $4. Atlanta, J. J. & S. P. Richards.

This new edition of Biddle's Materia Medica comes to us with the best of all indorsements—its large and rapid sale. Soft and flattering words fall pleasantly into the eager ears of the anxious author; fulsome praise and adulation even, may serve as a soothing balm to his oft wounded spirit; but after all this is but the breath of man, a thin vapory air. It costs nothing but a moderate compromise of sincerity and a little lowering of one's self-respect. When, however, the medical profession puts its hand deep into the breeches pocket and transfers its hard-earned dollars to the proffered palm of the publisher, it does homage to the author in a way that is both convincing and satisfying.

It has been the design of the author to present in his work a text-book for the student. It is brief, and yet sufficiently comprehensive. His style is clear and yet succinct. He covers the ground—covers it well, and cumbers his work with nothing superfluous.

The doses directed for a number of remedies are, we think, subject to criticism. Chloroform should not be directed in dose "f℥j, in sweetened water, or mucilage; to be repeated." We think it should not be stated: "The dose of chloral is twenty grains, which may be safely repeated every hour or two, till three doses have been taken, or sleep occurs." Gallic acid may be usefully employed in much larger quantity than "two to five grains every two or three hours." If iron-by-hydrogen be good, there is no need for "five to ten grains, three times a day."

